# Supplementary material for: Development and validity of the expectations of physiotherapists questionnaire on practice management software
Source: PeerJ. 2023 Oct 17;11:e16246. doi: 10.7717/peerj.16246 (PMC10588714; doi:10.7717/peerj.16246)
Supplement: Appendix S1 [file peerj-11-16246-s001.docx]

The following items were reformulated during Phase 2: Development of the items in the pretested with 11 physiotherapists in 2 focus groups. They are displayed both in the source language and translated into English. They are displayed both in the source language and translated into English. These were always preceded by the sentence: “El software deseable para su centro debería permitir...” (The desirable software for your center should allow...)

| Item prior to the pretest | Final items after the pretest |
| --- | --- |
| Posibilidad de que los pacientes puedan consultar y reservar cita previa online | Que sus pacientes puedan reservar online una cita |
| Acceder a cuestionarios y escalas funcionales | Acceder a cuestionarios (p.ej. EVA, NDI, DASH, SF-36) que puedan usarse para medir la funcionalidad de un paciente |
| La gestión del stock de productos utilizados en la clínica para su uso y/o venta (vendajes, cremas, theraband, etc.) | Generar informes del stock de fungibles del centro (p.ej. disponibles, consumos habituales) |
| Permitir el envío de comunicaciones para promociones, talleres, newsletters … | Enviar comunicaciones masivas (p.ej. para publicidad, promociones) a grupos de interés (corredores, por sexo, patología...) |

| Item prior to the pretest | Final items after the pretest |
| --- | --- |
| Possibility for patients to consult and book an appointment online | For your patients to book an appointment online |
| Access functional questionnaires and scales | Access questionnaires (e.g., VAS, NDI, DASH, SF-36) that can be used to measure a patient's functionality |
| The management of the stock of products used in the clinic for use and/or sale (bandages, creams, theraband, etc.) | Generate reports on the stock of consumables in the center (e.g., available, habitual consumption) |
| Allow the sending of communications for promotions, workshops, newsletters... | Send mass communications (e.g., for advertising, promotions) to interest groups (runners, by gender, pathology...) |
