# Supplementary material for: Development and validity of the expectations of physiotherapists questionnaire on practice management software
Source: PeerJ. 2023 Oct 17;11:e16246. doi: 10.7717/peerj.16246 (PMC10588714; doi:10.7717/peerj.16246)
Supplement: Appendix S2 [file peerj-11-16246-s002.docx]

This appendix shows the results of the complementary factor analysis for the two areas, both through maximum likehood factoring method (table 1 and 3) and through principal axis factoring method (table 2 and 4).

| **Table 1.** Factor analysis performed using maximum likelihood method of 12 items of the administrative activities area | | | | | |
| --- | --- | --- | --- | --- | --- |
|  | | **Value for factor** | | | |
| **Scales** | **Items** | **1** | **2** | **3** | **4** |
| Data entry and issuance of reports | Editable body charts | **0.799** | 0.199 | 0.115 | 0.111 |
|  | Digitalized patient-reported outcome measures | **0.783** | 0.258 | 0.106 | 0.127 |
|  | Templates for patients’ clinical reports | **0.677** | 0.214 | 0.205 | 0.104 |
|  | Editable templates for assessment | **0.627** | 0.290 | 0.155 | 0.235 |
|  | Templates for exercise programmes and recommendations | **0.582** | 0.186 | 0.209 | 0.177 |
| Monitoring quality of care | Quality measures | 0.258 | **0.881** | 0.182 | 0.220 |
|  | Healthcare activity reports | 0.364 | **0.742** | 0.196 | 0.221 |
|  | Patient safety reports | 0.303 | **0.713** | 0.137 | 0.131 |
| Digital health interventions | Videoconference | 0.195 | 0.120 | **0.956** | 0.179 |
|  | Chat | 0.252 | 0.266 | **0.635** | 0.133 |
| Patient portal | Online appointment booking | 0.144 | 0.163 | 0.123 | **0.877** |
|  | Consult scheduled visits | 0.254 | 0.235 | 0.185 | **0.677** |

| **Table 2.** Factor analysis performed using principal axes method of 12 items of the clinical care area | | | | | |
| --- | --- | --- | --- | --- | --- |
|  | | **Value for factor** | | | |
| **Scales** | **Items** | **1** | **2** | **3** | **4** |
| Data entry and issuance of reports | Editable body charts | **0.778** | 0.251 | 0.116 | 0.114 |
|  | Digitalized patient-reported outcome measures | **0.774** | 0.187 | 0.143 | 0.126 |
|  | Templates for patients’ clinical reports | **0.675** | 0.224 | 0.209 | 0.107 |
|  | Editable templates for assessment | **0.639** | 0.281 | 0.184 | 0.234 |
|  | Templates for exercise programmes and recommendations | **0.608** | 0.193 | 0.156 | 0.186 |
| Monitoring quality of care | Quality measures | 0.255 | **0.877** | 0.196 | 0.229 |
|  | Healthcare activity reports | 0.355 | **0.741** | 0.213 | 0.222 |
|  | Patient safety reports | 0.323 | **0.709** | 0.131 | 0.132 |
| Digital health interventions | Videoconference | 0.213 | 0.129 | **0.811** | 0.200 |
|  | Chat | 0.225 | 0.239 | **0.750** | 0.123 |
| Patient portal | Online appointment booking | 0.151 | 0.175 | 0.132 | **0.796** |
|  | Consult scheduled visits | 0.246 | 0.208 | 0.186 | **0.744** |

| **Table 3.** Factor analysis performed using maximum likelihood method of 14 items of the administrative activities area | | | | | | |
| --- | --- | --- | --- | --- | --- | --- |
|  | | **Value for factor** | | | | |
| **Scales** | **Items** | **1** | **2** | **3** | **4** | **5** |
| Issuance of routine documents | Templates for common documents | 0.266 | 0.185 | 0.103 | **0.880** | 0.154 |
|  | Automate the issuance of routine documents | 0.302 | 0.233 | 0.106 | **0.701** | 0.144 |
|  | Easily fill in and sign documents for patients and professionals | 0.363 | 0.156 | 0.215 | **0.587** | 0.133 |
| Data security | Saving and backup copies | **0.804** | 0.188 | 0.129 | 0.258 | 0.116 |
|  | Security measures against computer threats | **0.696** | 0.253 | 0.107 | 0.214 | 0.152 |
|  | Configuration of users and access permissions | **0.548** | 0.094 | 0.208 | 0.170 | 0.201 |
| Billing and accounting | Fees configuration | 0.218 | **0.883** | 0.148 | 0.181 | 0.190 |
|  | Flexibility in the application of fees | 0.249 | **0.635** | 0.270 | 0.271 | 0.223 |
|  | Allow different payment methods | 0.151 | **0.586** | 0.094 | 0.135 | 0.284 |
| Marketing strategies | Repository of standard messages | 0.193 | 0.226 | 0.078 | 0.106 | **0.839** |
|  | Allows mass mailings of communications | 0.067 | 0.260 | 0.036 | 0.264 | **0.588** |
|  | Links to external communication applications | 0.169 | 0.108 | 0.218 | 0.032 | **0.585** |
| Control stock supplies | Stock reports | 0.168 | 0.170 | **0.894** | 0.126 | 0.136 |
|  | Notifications to replenish consumables | 0.201 | 0.158 | **0.855** | 0.112 | 0.151 |

| **Table 4.** Factor analysis performed using principal axes method of 14 items of the administrative activities area | | | | | | |
| --- | --- | --- | --- | --- | --- | --- |
|  | | **Value for factor** | | | | |
| **Scales** | **Items** | **1** | **2** | **3** | **4** | **5** |
| Issuance of routine documents | Templates for common documents | 0.269 | 0.192 | 0.102 | **0.894** | 0.149 |
|  | Automate the issuance of routine documents | 0.316 | 0.223 | 0.109 | **0.689** | 0.142 |
|  | Easily fill in and sign documents for patients and professionals | 0.365 | 0.150 | 0.213 | **0.575** | 0.120 |
| Data security | Saving and backup copies | **0.799** | 0.187 | 0.131 | 0.257 | 0.124 |
|  | Security measures against computer threats | **0.711** | 0.253 | 0.105 | 0.191 | 0.158 |
|  | Configuration of users and access permissions | **0.543** | 0.106 | 0.201 | 0.183 | 0.171 |
| Billing and accounting | Fees configuration | 0.222 | **0.869** | 0.150 | 0.186 | 0.189 |
|  | Flexibility in the application of fees | 0.259 | **0.644** | 0.269 | 0.246 | 0.205 |
|  | Allow different payment methods | 0.152 | **0.601** | 0.089 | 0.127 | 0.275 |
| Marketing strategies | Repository of standard messages | 0.202 | 0.232 | 0.081 | 0.108 | **0.834** |
|  | Allows mass mailings of communications | 0.058 | 0.272 | 0.029 | 0.273 | **0.599** |
|  | Links to external communication applications | 0.161 | 0.099 | 0.218 | 0.020 | **0.588** |
| Control stock supplies | Stock reports | 0.172 | 0.170 | **0.901** | 0.121 | 0.138 |
|  | Notifications to replenish consumables | 0.202 | 0.162 | **0.849** | 0.116 | 0.152 |
