# Supplementary material for: Development and validity of the expectations of physiotherapists questionnaire on practice management software
Source: PeerJ. 2023 Oct 17;11:e16246. doi: 10.7717/peerj.16246 (PMC10588714; doi:10.7717/peerj.16246)
Supplement: Appendix S3 [file peerj-11-16246-s003.docx]

| **"Cuestionario sobre los atributos que debería ofrecer su software de apoyo a la gestión"** |
| --- |
|  |
| **Gestión clínica asistencial** |
| EL SOFTWARE DESEABLE PARA SU CENTRO DEBERÍA PERMITIR.... |
| 1 Acceder a plantillas de mapas corporales (u otras herramientas similares) que puedan usarse al explorar a un paciente |
| 2 Acceder a cuestionarios (p.ej. EVA, NDI, DASH, SF-36) que puedan usarse para medir la funcionalidad de un paciente |
| 3 Acceder a plantillas editables para emitir informes (p.ej. clínico-legales, mutuas) a empresas/clientes que lo solicitan |
| 4 Acceder a plantillas que definen la anamnesis y exploración a pacientes con patologías concretas |
| 5 Acceder a plantillas de ejercicios terapéuticos (o pautas) para seleccionar y personalizar un programa a su paciente |
| 6 Generar periódicamente informes de la calidad asistencial prestada en el centro (p.ej. resultados de encuestas de satisfacción) |
| 7 Generar periódicamente informes de la actividad asistencial prestada en el centro (p.ej. número de pacientes por patologías, nº de sesiones) |
| 8 Generar periódicamente informes de la seguridad de los pacientes en el centro (p.ej. incidencia de efectos adversos, caídas) |
| 9 Realizar videollamadas con pacientes u otros profesionales |
| 10 Chatear con uno o más pacientes en tiempo real |
| 11 Que sus pacientes puedan reservar online una cita |
| 12 Que sus pacientes puedan consultar online las fechas de las visitas pendientes o realizadas |
|  |
| **Gestión administrativa** |
| EL SOFTWARE DESEABLE PARA SU CENTRO DEBERÍA PERMITIR.... |
| 13 Acceder a plantillas de facturación o justificantes para emitir a empresas/clientes que lo solicitan |
| 14 Emitir fácilmente facturas, justificantes u otros documentos habituales |
| 15 Que el personal del centro y los pacientes puedan firmar documentos, consentimientos u otras autorizaciones (p.ej. mediante Tablet) |
| 16 Realizar guardados y copias de seguridad automáticas |
| 17 Disponer de medidas (p.ej. antivirus, cifrado) frente a amenazas informáticas |
| 18 Configurar los perfiles de acceso al software según el perfil de los trabajadores del centro |
| 19 Introducir todas las tarifas vigentes en el centro |
| 20 Aplicar excepcionalmente tarifas flexibles (p.ej. por un descuento) |
| 21 Ofrecer al paciente diferentes métodos de pago (p.ej. tarjeta, bizum, etc.) |
| 22 Acceder a mensajes-tipo editables para enviar a pacientes con un fin (p.ej. recordatorio, publicidad) |
| 23 Enviar comunicaciones masivas (p.ej. para publicidad, promociones) a grupos de interés (corredores, por sexo, patología...) |
| 24 Usar aplicaciones externas (p.ej. WhatsApp, Telegram) para enviar mensajes a pacientes |
| 25 Generar informes del stock de fungibles del centro (p.ej. disponibles, consumos habituales) |
| 26 Configurar avisos para reponer algún fungible (p.ej. porque su stock es escaso) |
