# Supplementary material for: Development and validity of the expectations of physiotherapists questionnaire on practice management software
Source: PeerJ. 2023 Oct 17;11:e16246. doi: 10.7717/peerj.16246 (PMC10588714; doi:10.7717/peerj.16246)
Supplement: Supplemental Information 5 [file peerj-11-16246-s005.docx]

**By area, the number of each item in the database is related to its corresponding subtheme.**

| **Subtheme** | **Category of coded statement** | **Item** |
| --- | --- | --- |
| Templates, digitalized tools and classification codes for data entry | Editable templates for assessment | 16 |
|  | Digital patient-reported outcome measures | 14 |
|  | Editable body charts | 15 |
|  | Classifications system for health problems and physiotherapy interventions | 20 |
|  | Customizable clinical history | 11 |
|  | Compilation of clinical records | 19 |
| Digitalized tools and templates for the issuance of individualized patient reports | Templates for reports | 18 |
|  | Exercise prescription software | 12 |
|  | Templates for exercise programs and guidelines | 17 |
| Digitalized measures to monitor quality of care | Patient-reported experience measures | 13 |
|  | Reports on the quality of care provided at the centre | 23 |
|  | Healthcare activity reports | 22 |
|  | Patient safety reports | 24 |
| Automatized reminders for patients and professionals within the scheduling agenda | Editable agenda | 1 |
|  | Send reminders to patients | 2 |
|  | Set alerts | 3 |
| Digital health interventions (DHI) | Videoconference | 25 |
|  | Chat | 26 |
|  | Corporate mail integrated | 27 |
| Patient portal | Online appointment booking | 31 |
|  | Consult scheduled visits | 32 |
|  | Access to support materials | 33 |

| **Subtheme** | **Category of coded statement** | **Item** |
| --- | --- | --- |
| Automatized issuance of routine documents | Automate the issuance of routine documents | 8 |
|  | Informed consent templates and other patient authorizations (e.g., data protection) | 6 |
|  | Templates for common documents (e.g., bills, attendance receipts) | 7 |
|  | Easily fill in and sign documents for patients and professionals | 9 |
|  | Digitalize external documents | 5 |
| Communication tools for marketing strategies | Links to external communication applications | 28 |
|  | Allow mass mailings of communications (advertising, promotions, etc.) | 30 |
|  | Repository of standard messages that can be modified | 29 |
| Flexible billing and automatized accounting of payments | Allow different payment methods | 42 |
|  | Fees configuration | 43 |
|  | Flexibility in the application of fees | 44 |
|  | Automatic synchronization of payment with accounting. | 45 |
|  | Financial reports | 46 |
| Automatized accounting of supplies to control stock | Stock reports (expenses, sales...) | 47 |
|  | Notifications to replenish consumables | 48 |
| Interoperability | Access from different devices | 37 |
|  | Ability to import and export data with ease | 38 |
| Data security tools to face failures, inadequate use and threats | Configuration of users and access permissions | 34 |
|  | Security measures against computer threats (anti-virus, encryption, etc.) | 36 |
|  | Saving and backup copies | 35 |
|  | Different data hosting options | 39 |
